# Chinese Paper-Cutting Style Transfer via Vision Transformer

**DOI:** 10.3390/e27070754

**Published:** 2025-07-15

**Authors:** Chao Wu, Yao Ren, Yuying Zhou, Ming Lou, Qing Zhang

**Affiliations:** 1 Engineering Training Center, Nanjing Vocational University of Industry Technology, Nanjing 210023, China; 2021101304@niit.edu.cn; 2 Academy of Art and Design, Anhui University of Technology, Ma’anshan 243002, China; renyao9188@foxmail.com (Y.R.); wuqishang008@outlook.com (Y.Z.); louming@ahut.edu.cn (M.L.)

**Keywords:** Chinese paper-cutting, style transfer, frequency-domain mixture encoder, feature contrastive learning

## Abstract

Style transfer technology has seen substantial attention in image synthesis, notably in applications like oil painting, digital printing, and Chinese landscape painting. However, it is often difficult to generate migrated images that retain the essence of paper-cutting art and have strong visual appeal when trying to apply the unique style of Chinese paper-cutting art to style transfer. Therefore, this paper proposes a new method for Chinese paper-cutting style transformation based on the Transformer, aiming at realizing the efficient transformation of Chinese paper-cutting art styles. Specifically, the network consists of a frequency-domain mixture block and a multi-level feature contrastive learning module. The frequency-domain mixture block explores spatial and frequency-domain interaction information, integrates multiple attention windows along with frequency-domain features, preserves critical details, and enhances the effectiveness of style conversion. To further embody the symmetrical structures and hollowed hierarchical patterns intrinsic to Chinese paper-cutting, the multi-level feature contrastive learning module is designed based on a contrastive learning strategy. This module maximizes mutual information between multi-level transferred features and content features, improves the consistency of representations across different layers, and thus accentuates the unique symmetrical aesthetics and artistic expression of paper-cutting. Extensive experimental results demonstrate that the proposed method outperforms existing state-of-the-art approaches in both qualitative and quantitative evaluations. Additionally, we created a Chinese paper-cutting dataset that, although modest in size, represents an important initial step towards enriching existing resources. This dataset provides valuable training data and a reference benchmark for future research in this field.

## 1. Introduction

The interdisciplinary convergence of artificial intelligence and computer vision has undergone transformative developments in artistic applications, marked by significant technological breakthroughs across core domains such as image processing, image detection, image classification, and image-style simulation. These advancements facilitate high-throughput processing of image datasets and the synthesis of cross-stylistic elements in innovative compositions. As a prominent frontier in computer vision, style transfer techniques [1] have emerged as a pioneering direction for computational artistic creation. Style transfer is a method in computer vision and computer graphics that generates new images by amalgamating the content of one image with the style of another. The objective is to create an image that retains the original’s content while applying the visual style of a different image, thus producing a more compelling artistic expression.

Review the development of style transfer, Gatys et al. were the first to use the VGG19 neural network model to extract content and style information and then iteratively generate stylized images from noisy images [2]. Building on Gatys’ work, researchers have proposed two major approaches to image-style transfer: image-based iteration and model-based iteration [3]. Image-based iteration involves refining the input image to carefully craft a unique artistic style. Contemporary investigations predominantly address three core methodologies: (a) maximum mean discrepancy-based style alignment [2,4], (b) Markov random field-driven style transformation [5], and (c) deep image analogy-enabled style fusion [6]. In parallel, iterative model refinement is achieved through gradient feedback mechanisms during network optimization. Notably, Johnson et al. pioneered a model-centered optimization strategy that employs feed-forward networks to effectively approximate solutions for style transfer optimization problems [7]. Similarly, Ulyanov et al. implemented analogous network architectures during training, employing generative models to enhance the quality of generated images [8]. For accelerating multi-style transfer, Zhang et al. developed a generative model with multi-style training capabilities [9].

Style transfer methodologies have progressed through three distinct evolutionary phases: initial implementations using conventional image processing techniques, subsequent developments with deep neural network-based approaches [2], and recent advancements incorporating Generative Adversarial Networks (GANs). Each technological advancement has considerably enhanced the integration mechanisms between content semantics and stylistic patterns. Image-style transfer methodologies have undergone substantial evolution, progressing from traditional techniques [10] to iterative optimization frameworks [4], and subsequently to computationally efficient feed-forward architectures [7,11]. Despite these advancements, simultaneous multi-style processing remains technically challenging. The development of generic style transfer mechanisms [12,13,14,15] has demonstrated effectiveness in addressing style distribution inconsistencies. On this basis, various innovative methods have emerged, such as ArtFlow [16] based on the flow model, comparative learning-based approaches [17,18], and feature stylization integrating attention mechanisms with Stable Diffusion Models (SDM) for creative generation. Significantly, emerging neural architectures—particularly Transformer-based frameworks—demonstrate profound potential in advancing style transformation methodologies. The advancement of image-style transfer technology has driven progress in artistic domains ranging from Western oil painting to traditional Chinese landscape painting. However, persistent challenges, including content-style imbalance, limited generalization capacity, insufficient multimodal coordination, and non-standardized evaluation metrics, continue to degrade the accuracy and stylistic fidelity of generated images [19,20,21]. These limitations are particularly evident in traditional art forms such as Chinese paper-cutting, where requirements for precise structural symmetry, intricate textures, and accurate edge segmentation remain insufficiently addressed by current style transfer methodologies.

In response to this challenge, this study presents an innovative hierarchical visual transformation framework specifically designed for the transfer of the Chinese paper-cutting style. The proposed method enhances the efficiency of transferring the distinctive symmetrical patterns characteristic of Chinese paper-cutting through a frequency-domain mixture block. Additionally, it improves the restoration of intricate features and the fidelity to the original style by maximizing mutual information between multi-level transformed features and content features. By integrating spatial-frequency information with reinforced symmetrical aesthetic structures, the method effectively preserves the intrinsic hollowed patterns and layered textures of paper-cutting art. The main contributions of this paper are summarized as follows:

- We propose a new style transfer method that accurately captures the unique symmetrical structures and aesthetic characteristics of Chinese paper-cutting art, achieving high-quality style transfer and efficiently generating artwork with a distinct paper-cutting style. In addition, we construct a dedicated Chinese paper-cutting dataset, providing rich training resources and benchmark data for future research, thereby filling an existing gap in the field.
- We design a frequency-domain mixture block that enhances the capture of both local textures and global symmetrical structures through Fourier convolutions, effectively distinguishing between style patterns and content details to improve style fidelity and visual coherence.
- A multi-level feature contrastive learning module is proposed, utilizing a visual Transformer encoder to extract multi-level style features, content features, and transformation features from the input image. By constructing a contrastive learning framework based on feature comparison, this module maximizes the correspondence between the transformed features and input features, thereby improving the fidelity of style transfer and content preservation.
- Extensive experiments show that, compared to existing advanced style transfer methods, the proposed method achieves superior overall performance in paper-cutting style transfer. It can quickly generate works that integrate the aesthetic and cultural value of Chinese paper-cutting art, providing new technical support for the digital creation of paper-cutting art.

## 2. Related Work

### 2.1. Characterization of Chinese Paper-Cutting

Given the technical constraints of existing style transfer methods, the extraction and selection of artistic style features for Chinese paper-cutting should focus on quantifiable and achievable attributes to facilitate subsequent implementation and quantitative comparative analysis. Additionally, the selected artistic features must ensure that the core characteristics of Chinese paper-cutting art are preserved, allowing users to accurately recognize that the style in the transformed image originates from Chinese paper-cutting. Based on this, Table 1 summarizes the feature extraction and selection results for the artistic style of Chinese paper-cutting.

Motivation: Traditional Chinese paper-cutting art exhibits unique frequency-domain characteristics, including repetitive high-frequency edges and highly structured low-frequency patterns due to its symmetrical and intricate nature. Motivated by this, we integrate the Fourier Transform into our architecture to explicitly separate and manipulate the frequency components of input features. This frequency-aware design allows our network to better preserve symmetric structures and fine-grained textures. Additionally, Pixel-wise Convolution (PConv) is employed to complement frequency information with enhanced spatial representations. While the individual components (e.g., PConv, FFT) have been used in prior works, their integration in a targeted manner for style transfer of Chinese paper-cutting images is novel and specifically designed to address the unique aesthetic requirements of this artistic domain.

### 2.2. Style Transfer

Style transfer has become an important research direction in the field of computer vision. This technology allows the fusion of features from a content image and a style image to generate a new image that combines both the style and content [18,22,23,24,25,26,27]. With continuous technological advancements, multiple paths for implementing style transfer have emerged [28,29,30,31,32,33,34]. Deng et al. [35] proposed an encoder-based method that constructs two different encoders to generate domain-specific sequences for content and style. Wang, P. et al. [36] introduced a simple and effective solution based on feature activation and SoftMax transformations. Feng, L. et al. [37] proposed a text-guided image-style method based on CNN and Restoemer’s dual-branch structure.

While traditional painting style transfer methods [15,16,17,18,38] have shown remarkable success, the inherent differences between Chinese paper-cutting art and Western oil painting—such as artistic expression, cutting techniques, and material carriers—make existing style transfer methods insufficient for converting the distinctive style features of Chinese paper-cutting art, such as intricate hollow patterns and well-defined lines. These methods are unable to produce satisfactory results when applied to Chinese paper-cutting.

### 2.3. Transformer

In recent years, Transformer models have made significant progress in the field of image-style transfer. Compared to traditional convolutional neural networks (CNNs), Transformers, through self-attention mechanisms, can capture long-range dependencies in images, thereby exhibiting stronger global modeling capabilities in style transfer tasks. For instance, Styleformer [39] was the first to introduce the Transformer into style transfer tasks, using a multi-scale attention mechanism to effectively merge the structural information of content images with the texture features of style images. Subsequently, Swin-Transformer [40] also made a successful application in image generation tasks, providing new insights for style transfer. Its sliding window attention mechanism significantly improves the quality of generated images while maintaining computational efficiency. Recently, StyTr2 [35] proposed a bidirectional Transformer-based style transfer framework, achieving more natural and diverse style transfer effects by modeling the feature distributions of both content and style simultaneously. S2wat [14] combined outputs from different window attention mechanisms, achieving powerful general-purpose style transfer. These works indicate that Transformer models possess considerable potential in the field of image-style transfer; however, significant challenges and research opportunities remain in their application to the style transfer of traditional Chinese art. Therefore, we further investigate the role of frequency-domain information in the style transfer process and propose a frequency-domain fusion encoder to enhance the refinement of style and content features for perceptually guided transfer.

## 3. Methodology

**Figure 1:**
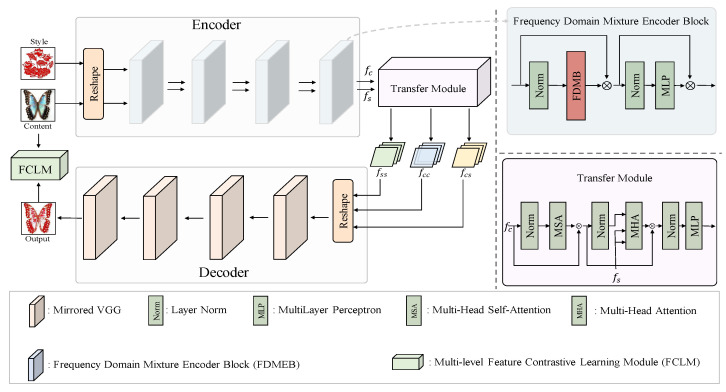
Illustration of the overall framework. Given a content image and a style image, the encoder produces corresponding features $f_{c}$ and $f_{s}$. These features undergo style transfer from $f_{s}$ to $f_{c}$ within the transfer module, yielding stylized features $f_{cs}$. Specifically, fcc and fss denote the features extracted by inputting two images with the same content or style, respectively. Subsequently, the decoder essentially mirrors the VGG network to decode the features into the output image. The output image is further refined by a feature contrastive learning module, which maximizes the correspondence between the transformed features and the input features across multiple levels, thereby enhancing the accuracy of style transfer.

In this paper, we aim to develop an effective style transfer network specifically designed for Chinese paper-cutting art, which is distinguished by its intricate patterns and symmetrical visual aesthetics. As illustrated in Figure 1, we first decompose the style and content images using multi-level window attention, enabling the network to capture both local details and global symmetrical structures. We then extract and fuse relevant features through a frequency-domain mixture encoder, which promotes the preservation of content semantics while enhancing the symmetrical consistency and stylistic representation. Following this process, a decoder is employed to generate an initial transformed image by decoding the fused features. As shown in Table 2, we summarize the spatial resolution and the number of channels for each stage of our backbone, which helps illustrate the multi-scale representation capability of the model. To further refine the output, we introduce a feature contrastive learning framework that maximizes the correspondence between transformed features and input features across multiple levels, thereby improving the accuracy of style transfer and reinforcing the symmetrical layout inherent in Chinese paper-cutting. The proposed method is detailed in the following sections.

### 3.1. Frequency-Domain Mixture Encoder

**Figure 2:**
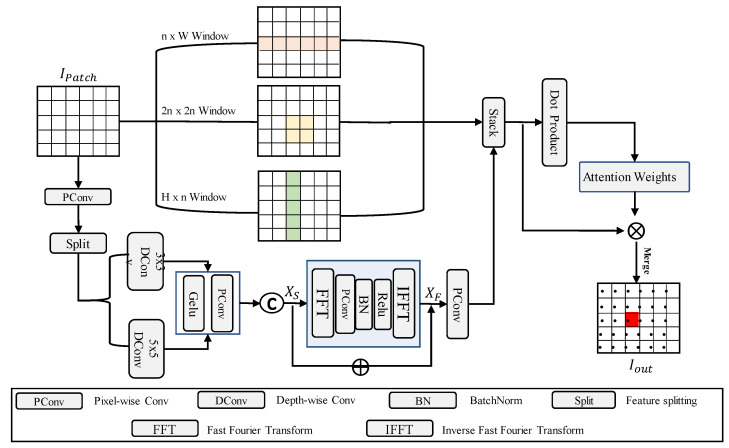
Illustration of the Frequency-domain Mixture Encoder Block.

We begin by resizing both the content and style images to 224 × 224. Following previous work [14], each image is partitioned into non-overlapping n×n patch blocks $I_{Patch}$, where *n* is set to 2. These patches serve as tokens in the Transformer architecture and are subsequently passed into the frequency-domain mixture encoder block. Specifically, the partitioned patches are embedded into three distinct attention windows: n×W, H×n, and 2n×2n, where padding operations are applied as needed to ensure divisibility and structural consistency. As illustrated in Figure 2, the Strip Window Attention module integrates these three directional attention mechanisms. The n×W and H×n attentions enhance long-range dependencies along horizontal and vertical axes, preserving global structural symmetry. Meanwhile, the 2n×2n window attention captures contextual information from the local surroundings, reinforcing fine-grained symmetrical patterns typically found in Chinese paper-cutting art. By combining these complementary attention windows, our model achieves a balanced integration of local details and long-range dependencies, while also maintaining the intrinsic symmetry of both content and style representations, thereby expanding the effective receptive field of the network.

(1) $$WA_{M\times M}\left(Q,K,V\right)=\mathrm{Softmax}\left(\frac{QK^{T}}{\sqrt{d}}+B\right)V$$

In this context, *Q*, *K*, and *V* refer to the query, key, and value matrices, respectively. The dimension d represents the matrix dimensions, and M×M indicates the use of multi-head self-attention with M×M window shapes. $B\in R^{M^{2}\times M^{2}}$ represents the relative positional bias, which is incorporated into the attention map computation of each head.

In addition, we perform Fourier convolution calculations on the input patches $I_{Patch}$. A space-frequency concatenation approach is employed to effectively process various textures and background information, facilitating the fusion of local and global features. The overall process is as follows: in the spatial domain operation, we first use Pixel-wise Conv (PConv) to enhance the input features and divide them into two groups to extract multi-scale local features. These features are then processed using 3×3 and 5×5 depth-separable convolutions. Afterward, the two feature groups are recombined using Pixel-wise Conv, which can be formally expressed as:

(2) $$X_{S}=PConv\left\{Gelu\left(DConv_{\Delta }\left(Split\left(PConv\left(I_{Patch}\right)\right)\right)\right)\right\}$$

Here, the $DConv_{\Delta }$ represents the spatial domain 3×3 and 5×5 depth-separable convolutions are represented. PConv refers to Pixel-wise Conv. Based on the features obtained above, we then perform frequency-domain operations. First, we apply the Fast Fourier Transform (FFT) to convert the features into high and low-frequency components in the Fourier domain, which are then concatenated. We then use a 1×1 kernel Pixel-wise Conv to perform the convolution operation. After adjusting, we effectively separate the Fourier domain. At this point, we apply the Inverse Fast Fourier Transform (IFFT) to convert the Fourier domain features back to the spatial domain. Finally, using a residual structure, we merge the features and send them into the Pixel-wise Convolution (PWConv) for channel compression, obtaining the final output:

(3) $$\begin{array}{cc} X_{H},X_{L} & =\mathrm{FFT}(X_{S})\\ X_{F} & =\mathrm{IFFT}(\mathrm{Gelu}\left(\mathrm{BN}\left(\mathrm{PConv}\left(\mathrm{Concat}\left(X_{H},X_{L}\right)\right)\right)\right))\\ I_{out} & =PConv(X_{S}+X_{F}) \end{array}$$

At this stage, we integrate the three window attention features with the previously obtained frequency-domain fusion features through a dynamic weighting strategy. Specifically, the four sets of features are tensor-stacked and subjected to a dot-product-based similarity computation to derive adaptive correlation weights. This dynamic fusion ensures that each feature contributes proportionally based on its relevance, resulting in a robust and expressive image encoding. The fused representation significantly enhances background-foreground differentiation while preserving intricate content details and maintaining visual symmetry across both spatial and frequency dimensions.

Transfer Decoder Module. Following the design of StyTr2 [35], our decoder adopts a Transformer-based architecture employing Multi-Head Self-Attention (MSA) at each layer to capture inter-feature dependencies and ensure symmetric information propagation. This is followed by Layer Normalization, a Feed-Forward Network (FFN), and a second Layer Normalization, which together refine the transferred features through a balanced and stable transformation pipeline. To reconstruct the final stylized image, we employ a pretrained VGG decoder, consistent with prior work, ensuring that the decoded output retains both the stylistic regularity and symmetrical structure characteristic of Chinese paper-cutting art.

### 3.2. Multi-Level Feature Contrastive Learning Module

**Figure 3:**
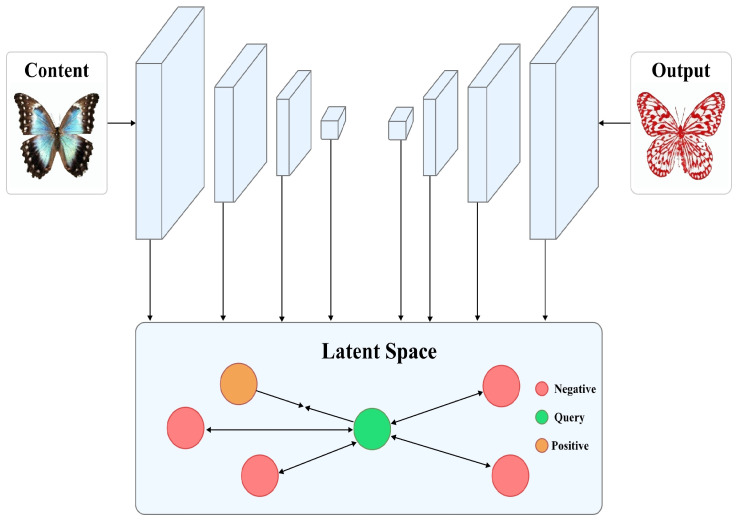
Illustration of the Multi-level Feature Contrastive Learning Module. The query indicates the output image features, positive indicates the corresponding content features, and negative samples indicate other content features.

Existing methods often lead to the loss of content details when dealing with the unique hierarchical and hollow characteristics of Chinese paper-cutting style transfer. Therefore, we have designed a feature contrastive learning module aimed at maximizing the mutual information between the output image and the content image. Its structure is shown in Figure 3.

The hollow characteristics of Chinese paper-cutting art necessitate precise contour delineation to distinguish the primary motif from the background, while preserving the structural integrity and artistic coherence of the design. This segmentation typically relies on smooth, flowing lines that form symmetrical and compact arrangements, embodying the balance between emptiness and substance. Therefore, during image processing, it is essential to effectively isolate the main subject from its surroundings. For instance, in the depiction of butterfly wings, the internal patterns should exhibit strong contrast against the wing’s body to accentuate the hollowed aesthetic unique to paper-cutting. Specifically, we feed the content image $I_{c}$ and transformed image $I_{cs}$ output from the decoder into VGG to extract multi-level features, which can be represented as:

(4) $${\left\{\phi_{l}\left(I_{c}\right),\phi_{l}\left(I_{c}s\right)\right\}}_{l}^{L},$$

Here, $\phi_{l}$ represents the *l*-th layer in the VGG network and *L* represents the total number of selected feature layers. In this experiment, *L* = 4. Then, to ensure consistent distribution of features at different levels, we first normalize the extracted feature maps:

(5) $$\hat{\phi_{l}}\left(I\right)={\frac{\phi_{l}\left(I\right)}{∥\phi_{l}\left(I\right)∥}}_{2},I\in \left(I_{c},I_{cs}\right).$$

Normalization eliminates the range differences in feature values, ensuring that similarity computations between different patches during contrastive learning are not biased by variations in feature magnitude. This step is crucial for preserving the structural balance across different spatial regions of the image. Additionally, average pooling is applied to downsample the feature maps at multiple scales, helping the model adapt to the multi-scale and hierarchical nature of the image features. Such processing is particularly important for Chinese paper-cutting, which often exhibits symmetrical compositions and fine structural patterns. From the downsampled feature maps, we randomly sample *N* feature patches to construct contrastive pairs. Specifically, the feature maps are first flattened into two-dimensional matrices, from which *N* patches are randomly selected. The resulting set of sampled patches is denoted as:

(6) $$P_{l}\left(I\right)=\left\{p_{i}|i\in S_{N}\left(I\right)\right\},$$

where $p_{i}$ represents the *i*-th patch and $S_{N}\left(I\right)$ denotes the index set of sampled patches from image *I*. This random sampling strategy maintains spatial diversity and promotes a balanced distribution of learned representations across the entire image space. To maximize the mutual information between the content image and the stylized output at corresponding spatial positions, while ensuring that the output image reflects distinct style characteristics, we adopt a contrastive learning framework based on the InfoNCE loss [41]. This loss not only pulls the output feature (query) $q_{i}$ closer to the corresponding content feature (positive) $p_{i}^{+}$ at the same location, but also pushes it away from unrelated features (negatives) $p_{i}^{-}$ sampled from other spatial locations in the content image. This dual objective enhances the model’s ability to preserve local details while promoting global symmetry. In this loss, query $q_{i}$ refers to the input image features, Positive $p_{i}^{+}$ refers to the content image features at the corresponding position, and Negative $p_{i}^{-}$ refers to the features from other locations in the content image. We define a contrastive learning loss function based on InfoNCE to pull the query closer to the Positive while pushing it farther from the Negative, as defined by the following specific form:

(7) $$L_{NCE}=-\frac{1}{N}\sum_{i=1}^{N}\log\left(\frac{\exp\left(q_{i}\cdot p_{i}^{+}/\tau \right)}{\exp\left(q_{i}\cdot p_{i}^{+}/\tau \right)+\sum_{j=1,j\neq i}^{N}\exp\left(q_{i}\cdot p_{j}^{-}/\tau \right)}\right)$$

where τ is the temperature parameter used to control the smoothness of the feature distribution.

To further enhance the loss function’s ability to capture features at different scales and layers, we extend this loss across multi-layer feature spaces. Specifically, we compute the contrastive learning loss independently for each feature layer and then compute a weighted sum:

(8) $$L_{FCM}=\sum_{l}^{L}\lambda L_{NCE}^{l}$$

where $L_{NCE}^{l}$ represents the feature loss at the *l*-th layer, and λ represents the corresponding weight coefficient. Low-level features focus more on edges and texture details, while high-level features capture semantic structural information. By fusing multi-level features, we can optimize both fine details and overall semantics comprehensively.

### 3.3. Network Training

We use content loss to measure the content difference between the stylized image $I_{cs}$ and the content image $I_{c}$, defined as:

(9) $$L_{content}=\sum_{l=4}^{5}{∥\Phi_{\mathrm{image}}^{l}\left(I_{cs}\right)-\Phi_{\mathrm{image}}^{l}\left(I_{c}\right)∥}_{2}$$

where $\Phi_{\mathrm{image}}$ represents the image encoder. In addition to the content loss, we use style loss to evaluate the perceptual effects of the style transfer process, defined as:

(10) $$\begin{array}{c} L_{content}=\sum_{l=1}^{5}{∥u\left(\Phi_{\mathrm{image}}^{l}\left(I_{CS}\right)\right)-u\left(\Phi_{\mathrm{image}}^{l}\left(I_{S}\right)\right)∥}_{2}\\ +{∥\partial \left(\Phi_{\mathrm{image}}^{l}\left(I_{CS}\right)\right)-\partial \left(\Phi_{\mathrm{image}}^{l}\left(I_{S}\right)\right)∥}_{2} \end{array}$$

where u(·) and ∂(·) represent the mean and variance of the features. At the same time, following previous works, we also adopt identity loss [15] to further preserve the structural features of the content image and the style features of the style image. This can be formalized as:

(11) $$L_{id}={∥|I_{CC}-I_{C}∥}_{2}+{∥|I_{SS}-I_{S}∥}_{2}$$

Here, $I_{CC}$ and $I_{SS}$ represent the output images stylized from two identical content (or style) images.

## 4. Experimental

### 4.1. Dataset

**Figure 4:**
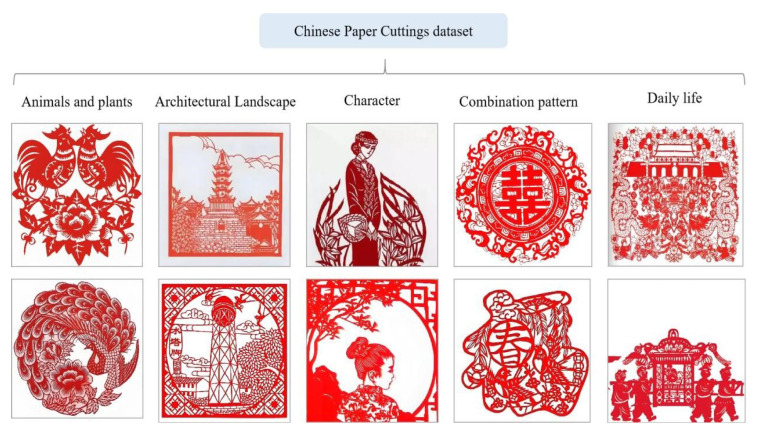
Sample Cases from the Chinese Paper-Cutting Dataset.

We collected 500 images of Chinese paper-cutting, which have unique artistic characteristics compared to other public datasets used for style transfer. For the data split, we selected 450 images for the training set and 50 images for the test set. Specifically, the dataset was constructed using a combination of offline photography and online image collection, focusing on representative styles of Chinese paper-cutting from different regions. The sources include Beijing paper-cutting from northern China, Nanjing styles from the Yangtze River Delta, Shaanxi paper-cutting from the northwest, and Foshan paper-cutting from the southern region. To ensure consistency and cultural authenticity, we primarily selected traditional red-colored paper-cut artworks, which are the most iconic and widely recognized in Chinese folk art. To enhance stylistic diversity, the dataset encompasses a broad range of common thematic categories, including flora and fauna, architectural landscapes, human figures, intricate composite patterns, and scenes depicting festive occasions and daily life. As shown in Figure 4, we show some samples of the dataset.

### 4.2. Implementation Details

We implemented our framework using PyTorch 1.7.1 on an NVIDIA RTX 3090 GPU. The initial learning rate for the network was set to 1 × 10 ^−4^, and we used the Adam optimizer with a batch size of 4. We trained for 40,000 iterations directly on the Chinese paper-cutting dataset and tested the model using images with different styles to evaluate its generalization capability and performance.

### 4.3. Comparative Experiments

In this section, we compare our method with previous state-of-the-art methods: Ghiasi et al. [42], CAST [18], StyTr2 [35], and S2WAT [14].

#### 4.3.1. Qualitative Comparison

**Figure 5:**
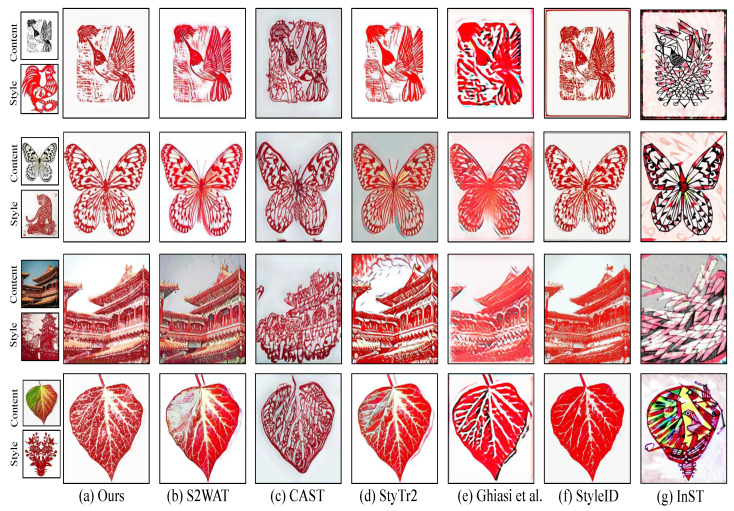
Comparison of Generated Results from Our Method and Other Methods. (**a**) Ours. (**b**) S2WAT [14]. (**c**) CAST [18]. (**d**) StyTr2 [35]. (**e**) Ghiasi et al. [42]. (**f**) StyleID [43]. (**g**) InST [23].

In this section, we compare our method with existing advanced methods, including those by Ghiasi et al. [42], CAST [18], StyTr2 [35], and S2WAT [14]. Figure 5 shows the inference results of these methods on the Chinese paper-cutting dataset, where the leftmost column shows the input content and style images, and the right columns show the inference results from each method. A comparison reveals that our method successfully transforms content images of various styles into works with the Chinese paper-cutting style. Specifically, as shown in the first three rows for butterfly images, our method demonstrates significant advantages over advanced methods like CAST and StyTr2. We successfully transform the butterfly’s texture and detailed features into the unique hollow style of paper-cutting, effectively suppressing color interference and noise. For the fourth row, depicting architectural style content images, S2WAT fails to effectively transform large areas of the background color, while CAST retains too many features from the style image. In contrast, our method significantly improves these issues by leveraging the multi-level feature contrastive learning module, effectively preserving the structural features of the content image. Moreover, the frequency-domain adaptive mixture block precisely captures and learns the unique style features of paper-cutting. Additionally, as shown in the last row, we tested our method on plant-style images, demonstrating good robustness and generalization ability, further verifying its applicability and reliability across different content styles.

#### 4.3.2. Quantitative Comparison

In this section, we evaluate our method using structural similarity SSIM, and two image perceptual metrics: LPIPS and FID. SSIM measures the preservation of image content structure, while LPIPS and FID assess the visual and stylistic quality of the generated images. As shown in Table 3, our method achieves the best performance across all three metrics. Notably, S2WAT exhibits a significant gap in content structure preservation, underscoring the influence of the unique and intricate style of Chinese paper-cutting on content details. Additionally, other methods demonstrate unsatisfactory perceptual evaluation results, highlighting the challenges associated with learning the style of Chinese paper-cutting. In contrast, our method displays superior performance in capturing the complexities of this art form while effectively retaining the structural details of the content image, resulting in satisfactory visual outcomes.

#### 4.3.3. User Ranking Experiment

In this section, we further validate our approach through a user voting experiment. We randomly selected three samples as cases and a uniform-style sample as a reference. Participants ranked the style transfer method proposed in this paper against four other methods (including Ghiasi et al.) across eight dimensions: smoothness of lines, flatness of pattern hollowing, clarity of pattern processing, color matching with red, material conformity to paper, cleanliness of background edges, content restoration, and similarity to the paper-cutting style. A total of 155 questionnaires were distributed, with 135 valid responses, yielding a validity rate of 87.1%. During the experiment, participants were asked to conduct both horizontal and vertical comparisons of the style input sample, the original pattern sample, and the style transfer output. They ranked the results from five methods based on each evaluation dimension. The best-performing method ranked first, and the worst-performing method ranked fifth. The calculation rule for the average comprehensive score is:

(12) $$Average\;Comprehensive\;Score=\frac{\sum Frequency\times weight}{Number\;of\;Respondents\;to\;this\;Question}$$

The weight is determined by the position of the option: 5 for the first position, 4 for the second, 3 for the third, 2 for the fourth, and 1 for the fifth. The final scores for all questionnaires were computed, and the detailed experimental results are shown in Table 4, Table 5 and Table 6.

From the user ranking results presented in the tables, it can be seen that our method performs excellently in style transfer across multiple different types of images. In all three categories (animal, plant, and pattern images), our method ranked first in the overall scores, further demonstrating the advanced nature and effectiveness of our approach for Chinese paper-cutting style transfer.

#### 4.3.4. Expert Scoring Experiment

To further validate the effectiveness and superiority of our Chinese paper-cutting style transfer method, we conducted an expert scoring experiment on the generated images from the sample cases. A total of 8 industry experts from 6 different organizations participated in this experiment. Before the experiment, we set a passing score threshold of 3.5, with an average score of 4 or higher considered excellent.

During the experiment, we randomly selected four groups of samples, each processed with style transfer, from different categories for expert evaluation. Experts scored each sample on six evaluation criteria, using a scale of 1 to 5, with 1 being the lowest and 5 being the highest. After all expert scores were collected, we computed the final average score for each category. The results are shown in Table 7.

Based on the data in the table, it is evident that our method achieves excellent results in generating Chinese paper-cutting style effects. The overall score for all sample categories exceeds 4, indicating that the results are considered excellent. Notably, smoothness of lines, clarity of pattern processing, material paper conformity, background edge cleanliness, and content restoration all scored above 4, with the red color matching and content restoration averaging above 4.5. These results further highlight the advanced nature of our approach.

### 4.4. Ablation Study

#### 4.4.1. Frequency-Domain Mixture Encoder Block

The quantitative ablation study results in Table 8 show a significant improvement in SSIM after introducing the frequency-domain mixture encoder block. This further verifies the module’s ability to effectively learn style features while retaining content information. As shown in Table 9, we analyzed the effects of different attention windows. The results indicate that the combination of directional windows (n×W and H×n) is crucial for capturing long-range symmetrical structures, while the 2n×2n window contributes more to preserving local details. Removing any of these windows leads to a significant decline in performance in terms of structural preservation and stylization fidelity. In addition, we separately evaluated the contributions of the spatial and frequency pathways. The results show that the spatial path enhances fine-grained details, while the frequency path better captures symmetrical layouts and repetitive textures. The fusion of both pathways brings complementary advantages, achieving an excellent balance between content preservation and stylistic consistency.

#### 4.4.2. Multi-Level Feature Contrastive Learning Module

**Figure 6:**
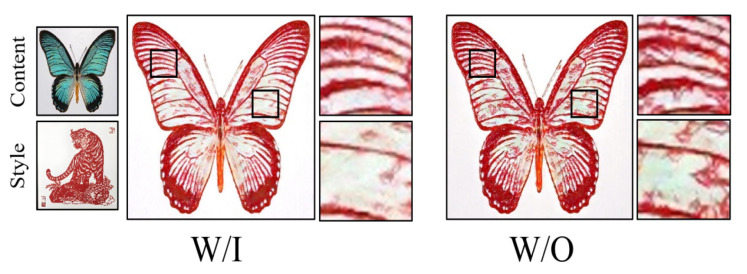
Results with and without the inclusion of the Multi-level Feature Contrastive Learning Module.

We designed the feature contrastive learning module to address the issue of content detail loss when transferring the Chinese paper-cutting style. To validate the effectiveness of this module, we conducted multiple ablation studies. As shown in Figure 6, we demonstrate the inference results with and without the inclusion of this module. The results clearly show that after incorporating this module, redundant background pixels in the output image are significantly reduced, and the original content information is better preserved while integrating the style. Additionally, as shown in Table 8, after introducing the multi-level feature contrastive learning module, our method showed significant improvement in both perceptual metrics: LPIPS decreased by 0.006, and FID decreased by 14.591. This indicates that the module performs well in generating generalized image details. The code and dataset is available at: https://github.com/ChaoWu6/ChinesePaperCutting (accessed on 20 May 2025).

## 5. Discussion

### 5.1. Practical Applications of Chinese Paper-Cutting Style Transformation

To further demonstrate the effectiveness and applicability of the Chinese paper-cutting style transfer method, we incorporated it into practical design applications and produced actual printed products for validation. Our approach conducts an in-depth analysis of the unique artistic features of Chinese paper-cutting, such as defined edges, hollow patterns, and the use of red, while integrating modern design concepts and technologies like printing and 3D printing. This enables the rapid application of paper-cutting elements in product design, providing new perspectives for existing product designs and further validating the effectiveness and broad applicability of our method.

**Figure 7:**
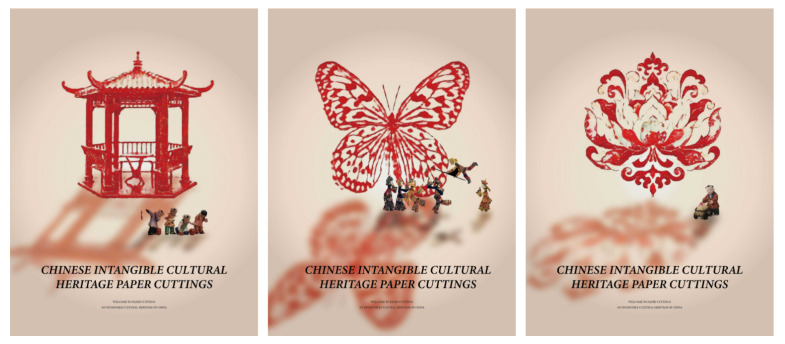
Application of Poster Design Based on the Transformation Method of Chinese Paper-Cutting Style.

**Figure 8:**
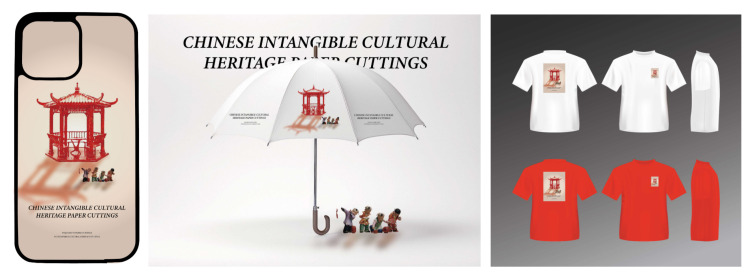
Design Applications of the Chinese Paper-Cutting Style Transfer Method on Phone Cases, Umbrellas, and Clothing.

**Figure 9:**
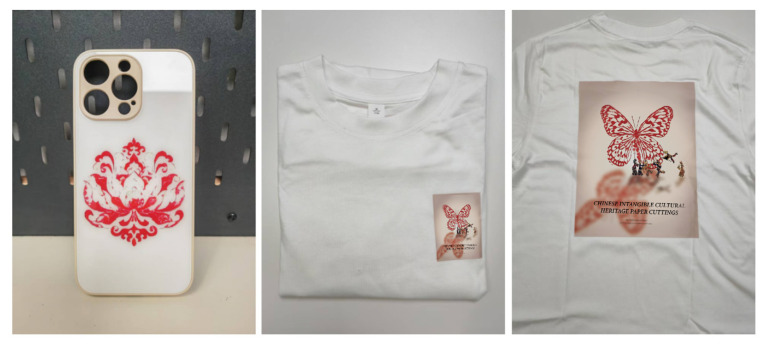
Display of actual printing effects on mobile-phone cases and clothing.

Firstly, we transformed the image samples into the Chinese paper-cutting style using the proposed methodology. Secondly, we disassembled and extracted elements from the transformed images, selecting multiple paper-cutting patterns to provide materials for subsequent designs. Then, we utilized Photoshop to complete the poster and related peripheral designs. As shown in Figure 7 and Figure 8, the generated design proposals effectively highlight the paper-cutting elements, reflecting the reliability of the method. Furthermore, as illustrated in Figure 9, we employed digital printing technology to produce printed designs for mobile-phone cases and T-shirts, thus realizing the practical production of the design proposals and allowing for a tactile wearing experience to better assess the overall effect. During the implementation process, the style transfer algorithm facilitated the rapid generation of design effects in the Chinese paper-cutting style, enabling its application in product design and manufacturing. The application of the style transfer method accelerated design efficiency and product rollout rates while significantly enhancing user perception.

In summary, the diverse applications of the Chinese paper-cutting style transformation method in the design field further validate the practical value of our approach and robustly demonstrate the potential of style transfer technology in the preservation and transmission of traditional paper-cutting art. This process of transmission and innovation not only contributes to the safeguarding of Chinese paper-cutting art but also serves as a reference for the preservation, transmission, and application of other forms of intangible cultural heritage.

### 5.2. Limitation

**Figure 10:**
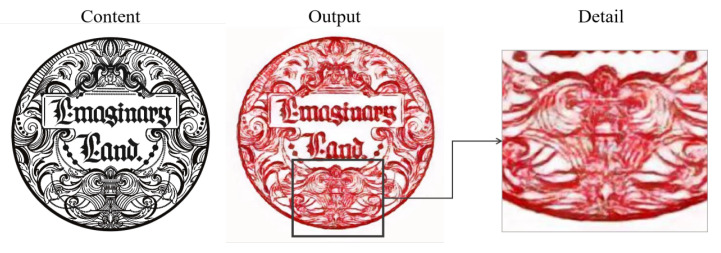
Limitations of our method when confronted with patterns with a high degree of symmetry.

**Figure 11:**
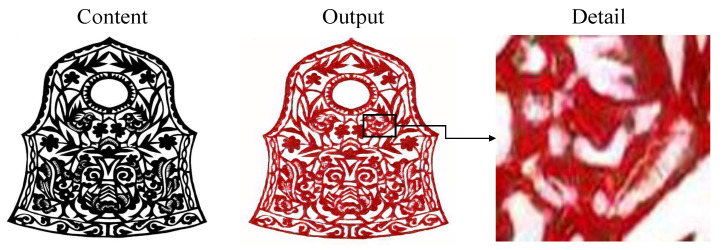
Limitations of our method when faced with a large number of openwork patterns.

Despite the significant progress made by our method in the field of transforming traditional Chinese paper-cutting art styles, several limitations remain that need to be addressed. Specifically, the current method has not fully optimized the style blending in transitional areas, which results in a lack of purity in the Chinese paper-cutting style, especially at the edges and fine details of the transferred artwork. Reducing the influence of these transitional areas would significantly improve the overall quality of style transfer, bringing the generated image closer to the artistic features of traditional Chinese paper-cutting. As illustrated in Figure 10, our method may struggle with accurately preserving symmetric structures. In some cases, structural distortion occurs in regions that should remain symmetric, leading to noticeable discrepancies between corresponding parts. Another limitation is observed in Figure 11, where intricate hollow patterns are present locally. The model tends to fill in these hollow areas, failing to retain the desired spatial openness that characterizes traditional Chinese paper-cutting.

These limitations are primarily due to two factors. First, while our model incorporates multi-level feature representations, it may lack explicit geometric constraints or inductive biases needed to enforce global symmetry. Second, the current feature fusion and reconstruction modules may not fully capture or reproduce fine-grained spatial variations, especially in regions with delicate cut-out structures.

In future work, we plan to introduce explicit symmetry-aware constraints, such as geometric priors or symmetry-preserving losses, to better guide the generation of symmetric patterns. Moreover, incorporating high-resolution feature refinement modules or attention mechanisms tailored for fine structure retention could enhance the reconstruction of intricate hollow regions. Expanding the dataset to include more diverse and complex patterns may also help the model generalize better to challenging cases.

## 6. Conclusions

Existing state-of-the-art style transfer methods often struggle to achieve satisfactory results when processing art forms like Chinese paper-cutting, primarily due to the unique hollow-out effects and layered compositional structures inherent to this craft. To address these challenges, we propose a novel style transfer network designed to accurately capture the distinctive artistic features of paper-cutting. Our approach achieves high-quality stylization, generating images that preserve both the strong sense of hierarchical composition and the authentic hollow patterns characteristic of this traditional art form. Specifically, our method first applies multi-level window attention to decompose the style and content images. This is followed by a frequency-domain adaptive hybrid encoding approach to extract and fuse relevant features, ensuring simultaneous retention of content details and style information. A decoder then performs initial decoding of the fused features to generate a preliminary stylized image. Finally, a feature contrastive learning framework is utilized to maximize the correspondence between transformed features and input features, guaranteeing that the output preserves the original content while fully exhibiting the distinctive hollow patterns and layered compositions of paper-cutting art. Rigorous validation through controlled comparisons, ablation studies, perceptual user evaluations, and expert assessments demonstrates the qualitative and quantitative superiority of the proposed framework. Empirical validation in design applications further confirms the practical utility of our methodology for paper-cutting stylization. This work establishes a methodological framework for adapting style transfer technologies to diverse forms of intangible cultural heritage, particularly with implications for preserving artistic authenticity while enabling creative reinterpretation.

## Figures and Tables

**Table 1:** Stylistic Characteristics of Chinese Paper-Cutting.

Style Dimension	Style Feature	Feature Description
Form Dimension	Smooth and Flowing Lines	Chinese paper-cutting involves using scissors to cut paper, resulting in clear and smooth edge lines that precisely outline the shapes. During style transfer, it is essential to maintain the fluidity and clarity of the lines in the image.
	Hollow Pattern on a Flat Surface	The hollow pattern is one of the most distinctive features of Chinese paper-cutting. Various images and patterns are presented in a flat form, with parts of the paper cut out to create transparent patterns. In style transfer, this should be represented by clear color blocks, removing unnecessary transitional color blocks, and creating rich visual effects with a flat representation.
	Clear Pattern Processing	Chinese paper-cutting emphasizes the display of clear and distinct shapes. Viewers should easily recognize the image shapes created by the paper-cutting. In style transfer, it is crucial to preserve the clarity and completeness of the image to ensure its recognizability.
Color Dimension	Red Color	Red is the most traditional and common color in paper-cutting, and the sight of a red pattern immediately evokes an association with paper-cutting. Therefore, during style transfer, it is necessary to remove any non-representative colors and adjust the color to red.
Material Dimension	Paper Texture	Chinese paper-cutting primarily uses paper as the medium. Therefore, during the transfer, the texture of the paper in the pattern should be maintained.
Craft Dimension	Clean Background and Edges	Chinese paper-cutting is crafted using scissors, resulting in backgrounds without irregular or rough edges. Therefore, during style transfer, it is necessary to ensure that the background remains free from color residue and that the edges are clean.

**Table 2:** Input and output dimensions of each module in the network.

Stage	Input Channels	Output Channels	Input Shape	Output Shape
Encoder	3	768	224 × 224	28 × 28
Transfer Module	768	768	28 × 28	28 × 28
Decoder	768	3	28 × 28	224 × 224
FDMB	C	2 × C	H × W	H/2 × W/2

**Table 3:** Qualitative results of style migration on the Chinese paper-cutting dataset.

Metrics	Ghiasi et al. [42]	StyTr2 [35]	CAST [18]	S2WAT [14]	StyleID [43]	InST [23]	Ours
SSIM	0.412	0.620	0.476	0.696	0.724	0.355	0.778
LPIPS	0.677	0.541	0.607	0.405	0.413	0.819	0.392
FID	476.126	427.414	416.446	263.655	239.620	244.011	230.165

**Table 4:** User Ranking Experiment Results for Style Transfer Methods on Animal Samples.

Content & Style	Ours	StyTr2 [35]	CAST [18]	S2WAT [14]	Ghiasi et al. [42]
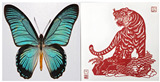	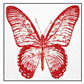	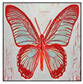	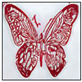	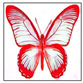	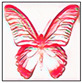
Smoothness of Lines	4.16	3.61	2.50	3.15	1.55
Flatness of Pattern Hollowing	3.97	3.25	3.01	3.04	1.71
Clarity of Pattern Processing	4.38	3.47	2.56	3.15	1.43
Red Color Matching	4.15	3.30	3.34	2.72	1.44
Material Paper Conformity	4.19	3.21	3.21	2.81	1.51
Background Edge Cleanliness	4.13	3.58	2.78	3.01	1.41
Content Restoration	4.45	3.51	2.53	2.90	1.56
Similarity to Paper-Cutting Style	4.19	3.27	3.15	2.76	1.57
**Overall Score**	**4.20**	**3.40**	**2.89**	**2.94**	**1.52**
**Overall Ranking**	1	2	4	3	5

**Table 5:** User Ranking Experiment Results for Style Transfer Methods on Plant Samples.

Content & Style	Ours	StyTr2 [35]	CAST [18]	S2WAT [14]	Ghiasi et al. [42]
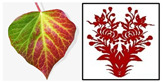	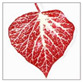	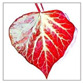	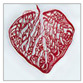	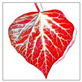	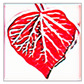
Smoothness of Lines	4.30	2.87	2.68	3.12	2.02
Flatness of Pattern Hollowing	3.96	2.41	3.24	2.96	2.41
Clarity of Pattern Processing	4.42	2.61	2.67	3.27	2.01
Red Color Matching	4.04	2.86	3.16	3.16	1.78
Material Paper Conformity	4.13	2.87	3.23	2.90	1.84
Background Edge Cleanliness	4.41	3.24	3.16	2.67	1.46
Content Restoration	4.16	3.41	3.2	2.29	1.93
Similarity to Paper-Cutting Style	3.85	2.41	3.70	2.80	2.19
**Overall Score**	**4.16**	**2.84**	**3.13**	**2.90**	**1.96**
**Overall Ranking**	1	4	2	3	5

**Table 6:** User Ranking Experiment Results for Style Transfer Methods on Pattern Samples.

Content & Style	Ours	StyTr2 [35]	CAST [18]	S2WAT [14]	Ghiasi et al. [42]
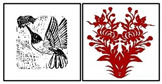	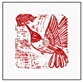	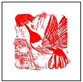	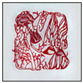	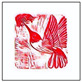	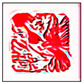
Smoothness of Lines	3.37	3.67	1.80	3.58	2.57
Flatness of Pattern Hollowing	3.39	3.46	3.27	2.87	2.01
Clarity of Pattern Processing	3.67	3.65	2.94	3.08	1.65
Red Color Matching	4.11	2.99	3.31	2.73	1.86
Material Paper Conformity	3.84	2.67	3.67	2.94	1.88
Background Edge Cleanliness	3.68	4.25	2.55	1.93	2.58
Content Restoration	3.82	3.46	2.21	3.58	1.90
Similarity to Paper-Cutting Style	3.97	2.73	3.95	2.74	1.59
**Overall Score**	**3.73**	**3.36**	**2.96**	**2.93**	**2.01**
**Overall Ranking**	1	2	3	4	5

**Table 7:** Expert Scoring Results.

TYPE	Samples			
	**Animal Samples**	**Plant Samples**	**Architectural Samples**	**Pattern Samples**
	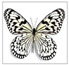	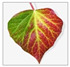	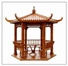	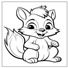
	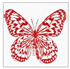	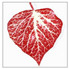	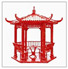	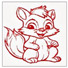
Smoothness of Lines	4.5	4.63	4.75	4.13
Flatness of Pattern Hollowing	4.25	3.88	4.63	4.25
Clarity of Pattern Processing	4.38	4.5	4.25	4.5
Red Color Matching	4.75	4.75	4.63	4.63
Material Paper Conformity	4.25	4.13	4.13	4.13
Background Edge Cleanliness	4.63	4.13	4.25	4.38
Content Restoration	4.75	4.63	4.75	4.63
Similarity to Paper-Cutting Style	3.88	3.88	3.88	4.0
**Overall Score**	**4.48**	**4.31**	**4.41**	**4.23**

**Table 8:** Ablation Study Results for Frequency-Domain Mixture Encoder Block (FDMB) and Melti-level Feature Contrastive Learning Module (FCLM).

Train Settings		Metrics		
**FDMB**	**FCLM**	**SSIM**	**LPIPS**	**FID**
w/o	w/o	0.699	0.405	256.333
w/	w/o	0.768	0.400	245.863
w/o	w/	0.745	0.399	241.742
w/	w/	0.778	0.392	230.165

**Table 9:** More ablation study results for frequency-domain mixture encoder block (FDMB).

Train Settings	Metrics		
**FDMB**	**SSIM**	**LPIPS**	**FID**
w/o Spatial branch	0.712	0.402	243.498
w/o Frequency branch	0.739	0.411	250.053
w/o n×W Window	0.767	0.395	234.402
w/o 2n×2n Window	0.755	0.397	238.173
w/o H×n Window	0.764	0.395	233.951
default	0.778	0.392	230.165

## Data Availability

The data presented in this study are available upon request from the corresponding author. The data are not publicly available due to copyright issues.
